# Cold sensitivity test for individuals with non-freezing cold injury: the effect of prior exercise

**DOI:** 10.1186/2046-7648-2-16

**Published:** 2013-05-01

**Authors:** Clare M Eglin, Frank StC Golden, Michael J Tipton

**Affiliations:** 1 Department of Sport and Exercise Science, Extreme Environments Laboratory, University of Portsmouth, Portsmouth PO1 2ER, UK

**Keywords:** Cold challenge, Blood flow, Skin temperature, Infrared thermography, Laser Doppler, Exercise

## Abstract

**Background:**

One of the chronic symptoms of non-freezing cold injury (NFCI) is cold sensitivity. This study examined the effects of prior exercise on the response to a cold sensitivity test (CST) in NFCI patients with the aim of improving diagnostic accuracy.

**Methods:**

Twenty three participants, previously diagnosed with NFCI by a Cold Injuries Clinic, undertook two CSTs. Participants either rested (air temperature 31°C) for approximately 80 min (prior rest condition (REST)) or rested for 30 min before exercising gently for 12 min (prior exercise condition (EX)). Following REST and EX, the participants placed their injured foot, covered in a plastic bag, into 15°C water for 2 min; this was followed by spontaneous rewarming in 31°C air for 10 min.

**Results:**

The great toe skin temperature (*T*_sk_) before immersion averaged 32.5 (3.4)°C in both conditions. Following immersion, the rate of rewarming of the great toe *T*_sk_ was faster in EX compared to REST and was higher 5 min (31.7 (3.4)°C vs. 29.8 (3.4)°C) and 10 min (33.8 (4.0)°C vs. 32.0 (4.0)°C) post-immersion. Over the first 5 min of rewarming, changes in the great toe *T*_sk_ correlated with the changes in skin blood flow (SkBF) in EX but not the REST condition. No relationship was observed between *T*_sk_ in either CST and the severity of NFCI as independently clinically assessed.

**Conclusions:**

Exercise prior to the CST increased the rate of the toe *T*_sk_ rewarming, and this correlated with the changes in SkBF. However, the CST cannot be used in isolation in the diagnosis of NFCI, although the EX CST may prove useful in assessing the severity of post-injury cold sensitivity for prognostic and medico-legal purposes.

## Background

Non-freezing cold injury (NFCI) is caused by prolonged exposure to cold and often wet environments. It is most commonly reported in the feet although the hands can also be affected. NFCI is usually associated with the military where it is the most common form of non-combat related injury in cold/temperate environments [1]. However, it may also occur in civilian populations undertaking recreational outdoor pursuits such as mountaineering and hill walking [2-4], diving [5], cycling [6], or in those working in hostile environments, e.g. fishing industry, agricultural workers, cold storage, etc. [7,8]. There have also been reports of NFCI in homeless individuals [7,9] and the elderly [10,11].

During World War II, Ungley identified four stages of NFCI in shipwrecked mariners [12,13]: in stage 1, during cold exposure, the tissue is ischaemic and numb; during rewarming (stage 2), the tissue becomes mottled blue and painful. In stage 3, the hyperaemic stage, which may last for up to 4 weeks, the tissue becomes swollen, red and hot with pain that may be persistent and severe. Stage 4, the chronic state, is characterised by cold sensitivity, numbness, hyperhidrosis and persistent pain that may last for many years. These chronic effects of NFCI may produce debilitating neurological problems including pain, paraesthesia, and even impaired neuromuscular function in severe cases that may have life-changing consequences for the individual. The cold sensitivity, which is an exaggerated vasoconstrictor response (in intensity and duration) to a cold stimulus, alone may cause protracted peripheral vasoconstriction leading to an increase in peripheral cooling and associated pain and numbness. Such consequences may affect employability status, increase living costs due to the requirement of specialised clothing and increased domestic heating, as well as restrict the individual’s participation in normal outdoor sporting or social activities. Cold sensitivity may also increase an individual’s risk of further cold injury on exposure to cold. As a consequence, in the military, many individuals diagnosed with NFCI are medically discharged [1]. Therefore, accurate identification of the severity of these long-term symptoms and the efficacy of their treatment is of importance in deciding future employability and treatment regimes in both military and civilian populations.

Cold sensitivity is assessed in cold injury clinics in the UK using a cold challenge test that involves the patient resting in an environment at 30°C for an hour before placing their injured limb in a plastic bag (to keep it dry) and immersing it in water at 15°C for 2 min. The limb is then allowed to rewarm spontaneously in air for 5 min. The assessment of the severity of the cold sensitivity is based on the skin temperature of the coldest digit measured using infrared thermography before immersion and after 5 min of rewarming [14,15]. The underlying principle behind the cold sensitivity test (CST) is that skin temperature reflects changes in local skin blood flow, and with a normal response, the peripheral vasculature will be vasodilated in a warm environment, vasoconstrict on exposure to cold and then quickly vasodilate on removal from the cold. Lower starting skin temperatures and slower rates of skin temperature rewarming indicate an exaggerated vasoconstriction and thus cold sensitivity. The diagnosis of NFCI is based on a combination of this cold sensitivity grading, the threshold of cold sensation measured using a thermal sensitivity tester, as well as a clinical consultation.

Studies in a control group of uninjured individuals have demonstrated that the response to the CST is variable both between and within individuals [16]. The studies indicated that some individuals, although not previously diagnosed with NFCI, may have a degree of cold sensitivity. It also raised questions about the specificity of the CST. The variability within uninjured individuals seen on repeat testing can be reduced by elevating core temperature slightly by performing gentle exercise prior to immersion [14]. This may also improve the sensitivity of the CST by removing any confounding influence of central vasoconstrictor drive, thus leaving the peripheral response to the CST as the primary determinant of the test result. This is desirable since NFCI is a local and not a systemic injury.

Preliminary unpublished pilot data on ten NFCI patients indicated that undertaking gentle exercise prior to the CST resulted in a cold sensitivity grading that was closer to an independent clinical assessment of NFCI [17]. Therefore, the aim of the present study was to expand upon these results using a larger cohort of individuals with NFCI. This study tested the hypothesis that, in comparison with resting in a warm environment, a short period of light exercise prior to undertaking a CST would accelerate the rate of return of skin temperature post-immersion as a result of increased blood flow to the injured extremity. To determine whether the severity of cold sensitivity can be used to diagnose NFCI, the results from both CSTs were compared to an independent clinical assessment of the severity of NFCI.

## Methods

### Participants

Twenty three individuals (17 African-Caribbean males, 4 Caucasian males, 2 African-Caribbean females, mean (SD) age, 29 (4) years) previously diagnosed with NFCI of the feet or hands and feet by a Cold Injuries Clinic gave their written informed consent to participate. The participants were referred to us as medico-legal cases for independent assessment of their NFCI, and therefore, no information on their original NFCI diagnosis by the Cold Injuries Clinic was available. Each undertook two CSTs of their injured limb (foot or foot and hand), one test in the morning and one in the afternoon. The order of the CSTs was randomized. The participants refrained from caffeine and smoking for 3 h prior to testing. The assessment was approved by a research ethics committee within the University of Portsmouth and was undertaken between January 2009 and 2011.

### Procedure

On arrival, the participants, wearing trousers and a long-sleeved top, entered a waiting room controlled at 31.1 (0.9)°C. Having removed their socks and shoes, they sat at rest for an average (SD) of 79 (19) min in the prior rest condition (REST) and for 31 (11) min in the prior exercise condition (EX). They then walked 15 m wearing slippers to another climatic chamber (30.6 (0.5)°C, relative humidity range 36% to 41%). In the REST condition, the participants sat for a further 10 min prior to the CST. In the EX condition, they performed light to moderate exercise for up to 12 min prior to the CST. The exercise consisted of either stepping at 22 steps.min^−1^ on a 20-cm step for 7–10 min (*n* = 3), exercising on an arm crank ergometer for 12 min at approximately 10 W (*n* = 1) or cycle ergometer for 12 min at 55 (20) W (*n* = 19). The mode, intensity and duration of exercise varied between participants due to varying fitness levels and peripheral neural pain experienced on exercising. A previous study in non-cold injured participants found increases in aural temperature of 0.3°C with these exercise intensities and ambient conditions [14]. Following the rest or exercise period, the participant sat reclined on a couch, and a multichannel laser Doppler probe (MoorLab System, Moor Instruments, Axminster, UK) was affixed using double-sided tape to the pad of the great toe of both feet to measure skin blood flow (SkBF). Two minutes of resting data were collected before commencing the CST.

During the CST, the injured foot was placed in a plastic bag (to keep it dry) and immersed to the level of the mid-malleoli for 2 min in stirred water at 15.0 (0.2)°C. The foot was then taken out of the water, the bag was removed, and the participant remained resting in a recumbent position on a couch for 10 min to allow spontaneous rewarming. Fourteen participants (ten African-Caribbean males, three Caucasian males and one African-Caribbean female) also had a CST performed on their injured hand (immersion to the level of the wrist) following the CST on their foot. The same procedure as that used with the foot was undertaken, with no further rest or exercise carried out between the tests.

Twenty two of the participants were assessed for NFCI whilst resting in a room at 31°C by a clinician with over 30 years experience of NFCI (one participant declined to see the clinician but undertook the two CSTs). The participants were interviewed to ascertain a detailed history including the circumstances leading to their NFCI, a description of the symptoms at the time of injury and the current condition/complaint. A physical examination was conducted to determine the appearance, trophic changes, degree of hyperhidrosis, peripheral pulse, capillary filling, sensation and gait of the injured limb. The severity of NFCI was based on the findings from the history and examination and was derived independently of the results from the CSTs.

### Measurements

Skin temperature (*T*_sk_) of the toe/finger pads of the immersed foot/hand was measured using a FLIR Systems A320G thermographic camera (UK) pointed at the soles of both feet/palm of both hands. Infra-red images, captured at 1 Hz to a remote computer, and *T*_sk_ were analysed at the following time points: prior to rest/exercise, prior to immersion, and every minute during rewarming. SkBF was recorded throughout the CST in arbitrary laser Doppler units (LDU) and minute averages were calculated for 2 min prior to immersion, during immersion and throughout the rewarming period. A biological zero Doppler measurement was taken on completion of each CST by manually compressing the arterial flow to the great toe/thumb.

As very similar *T*_sk_ profiles were observed for toes 2 to 5, the mean of these (*T*_t2–5_) and the *T*_sk_ of the great toe (*T*_Gt_) were analysed. *T*_t2–5_ and *T*_Gt_ prior to immersion, at 5 and 10 min of rewarming and the time taken to rewarm to 63% of the pre-immersion *T*_sk_ (calculated from the following equation *T*_63%_ = [*T*_PRE_ − *T*_IMM_] 0.63 + *T*_IMM_, where *T*_PRE_ is the pre-immersion *T*_sk_ and *T*_IMM_ is the *T*_sk_ immediately after immersion, [18,19]) were compared between REST and EX. *T*_sk_ of the thumb (*T*_th_) and the mean *T*_sk_ of the fingers (*T*_f_) were compared between REST and EX prior to immersion and at 5 and 10 min of rewarming.

### Data analyses

*T*_sk_ and SkBF data were analysed using Shapiro–Wilks and Kolmogorov–Smirnov tests of normality. To determine whether there was an order effect, *T*_sk_ before immersion and at 5 min of the first and second CST were compared using either an independent *t* test or a Mann–Whitney *U* test. Differences in skin temperature (*T*_Gt_, *T*_t2–5_, *T*_th_ and *T*_f_) and skin blood flow between REST and EX were analysed using a paired *t* test or Wilcoxon signed-rank test.

Spearman’s rank order correlation was used to investigate relationships between the severity of NFCI of the foot (from physical examination and personal history) and the *T*_sk_ obtained during the CSTs before immersion and at 5 and 10 min of rewarming. The relationship between change in *T*_Gt_ and change in SkBF over the first 5 min of rewarming was investigated using Spearman’s rank order correlation for EX and a Pearson’s product moment correlation for REST. Values are given as the arithmetic mean (standard deviation). Statistical significance was taken at the 5% level (*P* < 0.05).

## Results

On average, the participants were assessed 4.2 years (range 1 to 9.3 years) after their original injury. Typically, the injury occurred as a result of prolonged (5 days to 3 weeks) exposure to cold/freezing and often wet conditions. The severity of NFCI in the feet determined from the participants’ personal history and physical examination by the independent clinician was as follows: three uninjured, four mild, six mild/moderate, eight moderate, and one moderate-severe. The severity of NFCI in the hands was as follows: four uninjured, two mild, six mild/moderate, and one moderate.

No differences were found in *T*_Gt_ or *T*_t2–5_ between the first and second CST for either REST or EX conditions before immersion or at 5 min, indicating that the order of testing did not influence the *T*_sk_ observed.

**Figure 1:**
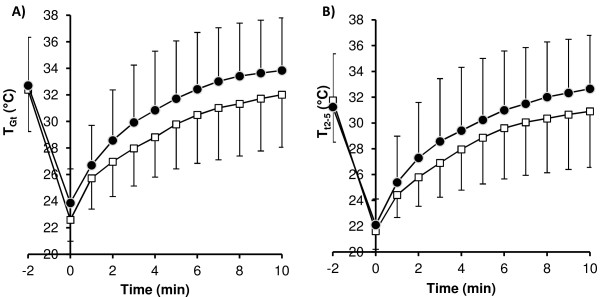
**Mean skin temperature (SD) of the toe pads during the REST and EX CSTs.** (**A**) *T*_sk_ of the great toe pad (*n* = 23). (**B**) Average *T*_sk_ of toes 2 to 5 (*n* = 23). REST (*open squares*) and EX (*filled circles*).

The *T*_sk_ of the toes before and during the REST and EX CST are shown in Figure 1. *T*_sk_ before immersion was similar in both conditions (Figure 1) as was the *T*_sk_ at 63% of pre-immersion values (*T*_Gt_: REST 28.7 (2.5)°C, EX 29.4 (3.0)°C; *T*_t2–5_: REST 28.0 (2.4)°C, EX 28.1 (2.8)°C, *P* > 0.05). The rate of rewarming of the great toe was faster in EX, with *T*_Gt_ reaching 63% of pre-immersion values sooner than during REST (178 (99) s vs. 249 (163) s, *t* = 2.148, *P* = 0.048). This faster rate of rewarming resulted in higher *T*_Gt_ at 5 and 10 min (Figure 1A). A faster rate of rewarming during EX was not as apparent in *T*_t2−5_, with only *T*_t2−5_ at 10 min being greater than REST (Figure 1B).

**Figure 2:**
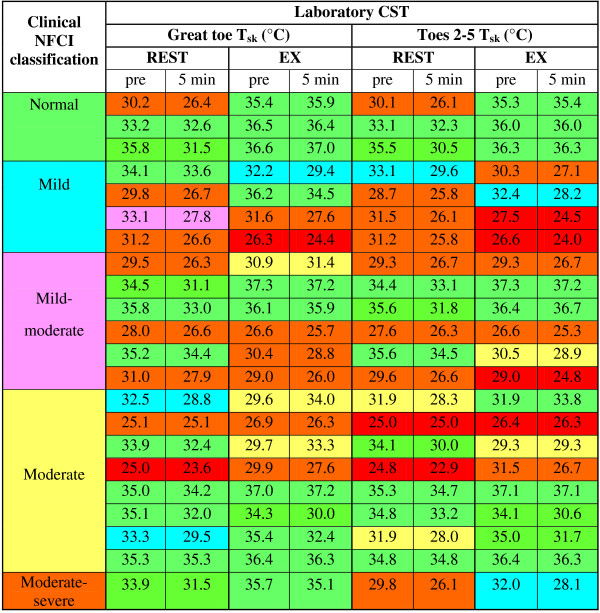
**Comparison of the skin temperature measured during the CSTs and NFCI severity assessed clinically.** The skin temperatures for the great toe and the mean of toes 2–5 for the REST and EX conditions are grouped according to the severity of NFCI clinically determined independently of the CST. Skin temperatures before immersion and that after 5 min of rewarming are given. The cold sensitivity grading resulting from these skin temperatures [14] are indicated by colours: normal = *green*, borderline = *light green*, mild = *blue*, mild-moderate = *pink*, moderate = *yellow*, moderate-severe = *orange*, severe = *red*.

The cold sensitivity gradings based on the participants’ *T*_sk_ before immersion and after 5 min rewarming are shown in Figure 2. No significant difference in grades was observed between the REST and EX conditions for either the great toe or the mean of toes 2–5. There was no relationship between the classification of NFCI made independently by the clinician and the results of the laboratory CST test (Figure 2).

**Figure 3:**
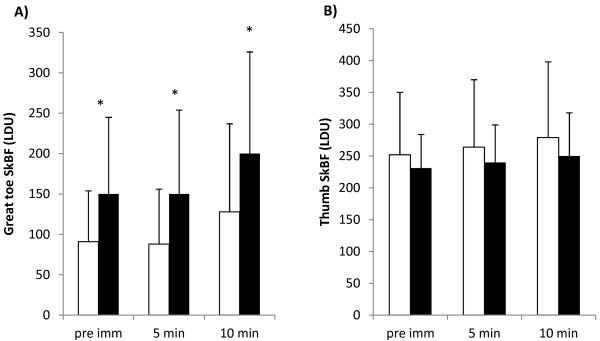
**Mean skin blood flow (SD) during the REST and EX CST.** Each *bar* represents the average SkBF over a minute period. REST (*open bars*) and EX (*filled bars*). *Asterisk* indicates a significant difference between REST and EX (*P* < 0.05). (**A**) Mean SkBF of the great toe pad (*n* = 23). (**B**) Mean SkBF of the thumb (*n* = 14).

Absolute SkBF in the great toe was higher in the EX condition compared to the REST condition before immersion and during rewarming (Figure 3A). When the SkBF was normalised to the resting pre-immersion levels, no differences were observed between CST at either 5 min (REST 96 (47)%, EX 110 (55)%, *P* > 0.05) or 10 min (REST 141 (68)%, EX 153 (90)%, *P* > 0.05).

**Figure 4:**
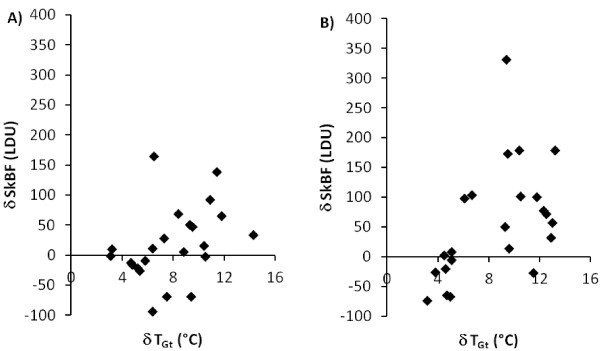
**Relationship between the change in great toe skin blood flow and the change in skin temperature.** The change was calculated over the first 5 min of rewarming during the REST and EX CST. (**A**) REST CST (*r* = 0.368, *P* = 0.84). (**B**) EX CST (*r* = 0.598, *P* = 0.003).

The change in *T*_Gt_ was correlated with the change in great toe SkBF over the first 5 min of rewarming in the EX (*r* = 0.598, *P* = 0.003) but not REST condition (Figure 4).

**Figure 5:**
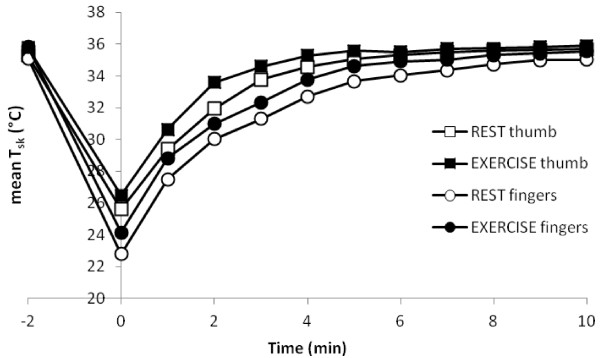
**Average mean skin temperature of the thumb and finger pads during the REST and EX CSTs (*n*= 14).**

*T*_sk_ of the thumb and fingers before and during the REST and EX CST are shown in Figure 5. No differences were observed between the REST and EX conditions for either *T*_sk_ or SkBF (Figures 3B and 5). No relationship was observed between the change in *T*_th_ and thumb SkBF over the first 5 min of rewarming in either CST.

Based on *T*_sk_ of the thumb before immersion and after 5 min rewarming, all but one individual were graded as having a normal response to the CST (however, it was not the same individual for both tests: in the REST condition, one participant was graded as moderate/severe, and in the EX condition, another participant was graded as borderline). Based on the mean *T*_sk_ of the fingers, two individuals were graded severe and one as borderline in the REST condition and one as borderline and one as mild-moderate in the EX test; the rest were graded as normal.

## Discussion

The diagnosis of NFCI in patients suffering overt neurological changes is relatively straightforward, but quantifying the degree of residual cold sensitivity from the patient’s history alone can be difficult. Yet, for prognostic and medical disposal reasons, an accurate assessment of the degree of sensitivity is important, especially in medico-legal cases. The long-term sequelae of NFCI is very variable with about 60% of individuals reporting cold sensitivity and 70% pain [15]. This was supported in the current study by the range in severity of NFCI and cold sensitivity in individuals who were all seeking compensation for their cold injury. In addition, severity of symptoms during and shortly after the original cold exposure does not necessarily predict the long-term symptoms [13].

This study was undertaken to compare two methods of assessing the cold sensitivity of individuals with NFCI in an endeavour to improve the diagnostic capability of the test. It was hypothesised that compared to the REST condition, performing mild exercise before the CST (EX) would remove any residual central vasoconstrictor tone thus increasing the rate of rewarming in the limbs of individuals with mild to moderate cold sensitivity. It was anticipated that this would result in a better correlation with the independent clinical assessment of NFCI, which was based on the symptoms, history and examination.

The EX CST resulted in a faster rate of rewarming and consequently a higher *T*_sk_ at 5 and 10 min compared to the REST condition (Figure 1). However, this did not translate into a statistically significant difference in the cold sensitivity grading between the two CST. This was due to the strong effect of the starting *T*_sk_ on the classification given; a *T*_sk_ above 32°C is classified as normal to mild/moderate, whereas a starting *T*_sk_ below 32°C is, at best, graded as moderately cold sensitive [14]. Since no difference in starting *T*_sk_ was observed between the CSTs (Figure 1), it is not surprising that the derived cold sensitivity gradings were similar. This would suggest that the pre-test skin temperatures observed after equilibration in an ambient temperature of 30°C do not accurately reflect the ability of the local tissue temperatures to recover following the cold exposure.

Previous unpublished studies have failed to identify a relationship between skin temperature and blood flow during a CST [14,17]. This raised issues regarding the specificity of the CST, as it would be expected that changes in skin temperature would be a result of changes in skin blood flow. It has been hypothesised that this lack of relationship may be due to the presence of a variable degree of central vasoconstrictor tone [14]. In the current study, a moderate relationship (*r* = 0.598) was found between the change in SkBF and *T*_Gt_ during the first 5 min of rewarming in the EX but not the REST condition (Figure 4). This suggests that a slight elevation of core temperature through gentle exercise removes this variable central vasoconstrictor tone, thus isolating the peripheral response to the local cold challenge. This should be regarded as a benefit, given that the test should be solely evaluating the effect of a cold stimulus on the local circulatory response in a region with a local injury. However, no correlation was found between the severity of NFCI as assessed through the personal history and clinical examination and the skin temperature during either CST (Figure 2). The NFCI clinical diagnosis is based on the detailed history of the original injury (i.e. presence/absence of swelling, blistering or loss of nails) and current condition (e.g. pain, peripheral thermal perception at normal room temperature, trophic changes and any gait problems).

The majority of our participants were of African-Caribbean origin and reflect the increased prevalence of NFCI reported in this ethnic group compared to Caucasians in both the British [20] and the US army [21]. The reason for this increased risk of NFCI in African-Caribbeans is unclear. However, there do appear to be ethnical differences in the uninjured response to a cold challenge, with African-Caribbeans showing lower finger *T*_sk_ and fewer cold-induced vasodilatations (CIVDs) compared to their Caucasian counterparts [22-24]. The occurrence of CIVDs during a cold challenge when the body is normothermic is thought to provide protection against profound peripheral cooling and thus reduce the risk of cold injuries [25]. Indeed, it has been proposed that repeated cold water immersions of the extremities results in a local cold adaptation characterised by a higher *T*_sk_, more CIVDs and less pain which maintains manual performance as well as reducing the risk of NFCI [26]. Therefore, a more pronounced and prolonged vasoconstriction with fewer CIVDs during a cold challenge may make African-Caribbeans more susceptible to NFCI. A comparison between the responses of the African-Caribbean and Caucasian participants (and similarly between males and females) was not possible in the current study due to the varying circumstances and time since their initial injury and the range in severity of the subsequent NFCI.

## Conclusions

It is concluded that gentle exercise prior to undertaking a CST results in a faster rate of *T*_sk_ rewarming and shows a better relationship between the SkBF and *T*_sk_ in individuals previously diagnosed with NFCI. Thus, the EX CST may be a useful tool for identifying cold sensitivity. The *T*_sk_ measured during either CST did not correlate with the severity of NFCI assessed clinically, and therefore, a cold sensitivity test should not be used, in isolation, to diagnose NFCI.

## Abbreviations

CST: Cold sensitivity test; EX: Prior exercise condition; LDU: Laser Doppler units; NFCI: Non-freezing cold injury; REST: Prior rest condition; SkBF: Skin blood flow; Tf: Finger skin temperature; TGt: Great toe skin temperature; Tsk: Skin temperature; Tt2–5: Mean skin temperature of toes 2 to 5; Tth: Thumb skin temperature; T63%: Time taken to rewarm to 63% of the pre-immersion *T*_sk_.

## Competing interests

The authors declare that they have no competing interests.

## Authors’ contributions

All the authors contributed to the design of the study and writing the protocol. MT initiated the project and provided funding. FG undertook the clinical assessments, and CE was responsible for the data collection during the CST and analysis of the data. All authors contributed to the writing of the paper and read and approved the final manuscript.
